# Virtual reality simulation for tracheostomy emergencies: a randomized educational intervention

**DOI:** 10.1093/atsscholar/aapag023

**Published:** 2026-05-23

**Authors:** Jordan W Talan, Mark H Adelman, Molly Forster, Brian Reuland, Brian Kaufman, Ali Hafiz, Sunil S Nair, Violet E Kramer, Jonathan S Mendelson, Anthony Andriotis

**Affiliations:** Department of Medicine, Division of Pulmonary and Critical Care Medicine, New York University Grossman School of Medicine, New York, NY, United States; Department of Medicine, Division of Pulmonary and Critical Care Medicine, New York University Grossman School of Medicine, New York, NY, United States; Department of Medicine, Division of Pulmonary and Critical Care Medicine, New York University Grossman School of Medicine, New York, NY, United States; Department of Medicine, Division of Pulmonary and Critical Care Medicine, New York University Grossman School of Medicine, New York, NY, United States; Department of Medicine, Division of Pulmonary and Critical Care Medicine, New York University Grossman School of Medicine, New York, NY, United States; Department of Anesthesiology, New York University Grossman School of Medicine, New York, NY, United States; Department of Medicine, Division of Pulmonary and Critical Care Medicine, New York University Grossman School of Medicine, New York, NY, United States; Department of Medicine, Division of Pulmonary and Critical Care Medicine, Jefferson Health, Abington, PA, United States; Department of Medicine, Division of Pulmonary and Critical Care Medicine, RWJBarnabas Health, Monmouth Medical Center, Long Branch, NJ, United States; Department of Medicine, Division of Pulmonary and Critical Care Medicine, Hartford HealthCare Medical Group at St. Vincent’s Medical Center, Bridgeport, CT, United States; Department of Medicine, Division of Pulmonary and Critical Care Medicine, New York University Grossman School of Medicine, New York, NY, United States

**Keywords:** medical education, graduate medical education, simulation training, virtual reality

## Abstract

**Background:**

Despite a high incidence of tracheostomy-related airway complications with potentially life-threatening implications, nonsurgical tracheostomy first responders receive limited formal education on the management of tracheostomy emergencies. While the United Kingdom has developed multidisciplinary guidelines and education for tracheostomy emergencies, such programs have not been widely implemented in the United States.

**Objective:**

We evaluated the feasibility and effectiveness of an immersive virtual reality (VR) simulation training as a potential generalizable and scalable approach to tracheostomy-related emergency training.

**Methods:**

Over the academic year 2023-2024, critical care fellows were randomly assigned to participate in tracheostomy emergency training either via immersive VR simulation or via small group discussion sessions facilitated by expert faculty. After each case-based educational intervention, participants were asked to manage 4 simulated tracheostomy-related emergencies involving common tracheostomy complications. Fellow performance was evaluated using a purpose-built task trainer. Three independent and blinded graders completed fellow scoring using a checklist assessment for which validation evidence was also collected. Fellows received pre- and postintervention surveys to measure attitudes toward VR training.

**Results:**

Of 27 eligible fellows, 19 participated in the study, managing 76 simulated tracheostomy emergencies. There were 10 fellows in the VR arm and 9 fellows in the small group arm. Of a total possible 26 points on the checklist assessment, fellows in the VR group scored an average (SD) of 18.03 (3.39) compared to the small group score of 16.96 (4.41) (*P* = .558). Surveys indicated improvements in fellow confidence after the training and high levels of acceptance of the VR curriculum.

**Conclusions:**

An immersive VR educational intervention for the management of tracheostomy-related emergencies was feasible and well received by learners. There was no significant difference in post-training checklist assessment scores between the VR and small group participants, suggesting noninferiority of the VR intervention and contributing validation evidence to our task trainer simulation assessment.

## Introduction

Despite a high incidence of tracheostomy-related airway complications with potentially life-threatening implications, nonsurgical tracheostomy first responders receive limited formal education on the management of tracheostomy-related emergencies. Immersive virtual reality (VR) simulation has the potential to be a cost-effective and scalable method to provide education in this subject, but its effectiveness on educational outcomes, including among a learner population of critical care trainees, has not been adequately investigated.

### Tracheostomy emergencies

Tracheostomy is performed more than 100 000 times annually in the United States, and its incidence is increasing.^1^ Patients with tracheostomy tubes are at high risk for complications that can be life-threatening, especially if not recognized in a timely manner and managed appropriately.^2^^,^^3^ The Royal College of Anaesthetists studied serious airway complications from all National Health Service (NHS) hospitals over 1 year in their Fourth National Audit Project.^4^^,^^5^ Of all events in the intensive care unit (ICU), the most common complications occurred in patients with existing tracheostomy tubes, and 61% of these complications resulted in death or permanent neurologic injury. Aside from patient-related factors, limitations in education and training were the most frequent contributory factors, playing a role in 58% of cases. This study highlighted a need for increased training in tracheostomy emergency management, a gap in competency that has now been echoed throughout the literature, particularly for nonsurgical first responders.^6^ Despite studies showing that educational interventions can improve confidence and knowledge in this area, there are no defined best practices or well-validated interventions for tracheostomy emergency education in the United States.^7-9^

To address the gap in tracheostomy emergency training throughout the United Kingdom, the National Tracheostomy Safety Project (NTSP) published multidisciplinary guidelines and educational resources for the management of tracheostomy and laryngectomy emergencies.^10^ In a study of NTSP resources including e-learning modules, educational videos, and multidisciplinary courses from the NTSP among 4 NHS hospitals, implementation was associated with significant reductions in reported tracheostomy-related safety incidents.^11^ Despite the success of this program, similar programs have not been widely implemented in the United States. Formal training on tracheostomy emergencies, even among anesthesia training programs, is frequently missing from the established curriculum.^12^^,^^13^ To best serve our patients with tracheostomies, we need educational interventions that are scalable to large numbers of trainees, easily reproducible, and have validation evidence among nonsurgical tracheostomy emergency first responders.

### Immersive VR simulation

Immersive VR simulation allows learners to practice clinical skills in a computer-generated environment, affording the opportunity to engage with surroundings in an authentic manner.^14^ Virtual reality offers many advantages as a simulation modality, including the ability to facilitate distance learning and to simulate cases that would be challenging to approximate using mannequins or task trainers.^14^ In comparison with high-fidelity mannequin simulation, VR has shown improved or comparable educational outcomes for technical and nontechnical skill acquisition.^15-18^ Furthermore, VR can be more cost effective and less resource intensive.^18^ Virtual reality is particularly promising as an educational modality to teach tracheostomy emergency management because it is easily repeatable and can be scaled to a large population of learners in geographically diverse settings.^19^

While VR simulation for teaching tracheostomy emergency management is appealing in theory, there remains a relative paucity of research characterizing learning outcomes using this modality. In 2022, Chiang  et al.^20^ published a trial in which 60 healthcare providers, including physicians, nurses, and respiratory therapists, were randomly assigned to receive either text-based or VR simulation education on tracheostomy care. Educational content predominantly focused on routine tracheostomy care but also included emergency scenarios such as aspiration, desaturation, dislodgement, and tube occlusion. Learners in the VR arm reported improved self-efficacy outcomes, including familiarity, confidence, and reduced anxiety around tracheostomy management, as well as higher satisfaction with VR training materials. Preceptors audited trainees’ performances via a checklist-based assessment for nonemergent care only, and the VR group had significantly higher scores.^20^ Further contribution to this literature was published recently by Abbas  et al.^21^ in the United Kingdom, who randomly assigned 37 learners to participate in either a face-to-face course mirroring the established procedures of the NTSP, an in-person immersive VR version of that material, or a remote VR experience. There was no significant difference in the primary outcome, which was scored on a written knowledge assessment. All participants resolved the primary clinical problem in both groups, but simulation outcomes data were limited to time-to-completion, with the face-to-face group performing significantly faster.^21^

To take the next step and further support the validity of immersive VR simulation training for tracheostomy emergencies, we need performance-based and well-validated assessments that can characterize associated educational outcomes among generalizable populations. We aim to contribute to this literature by describing the development and assessment of an immersive VR curricular intervention to teach emergency tracheostomy management to adult critical care fellows.

## Methods

We developed an immersive VR educational intervention to teach tracheostomy-related emergency management and conducted a randomized educational intervention of critical care fellows assigned to either VR simulation or small group discussion.

### Fellow recruitment and participation

Over the academic year 2023-2024, critical care fellows at our large academic medical center were recruited for participation via e-mail. Fellows were randomly assigned to participate in either a VR simulation training session or small group discussion. Fellows with any history of VR tracheostomy training were excluded.

The VR group participants each underwent a 15-minute orientation to the VR environment called Sandbox during which they were placed into the same VR intensive care room in which the cases would take place. There, participants had the opportunity to learn how to pick up and use objects with the VR hand controllers, practice interacting with the virtual patient, and discover where necessary airway equipment was stored. The goal was to eliminate this cognitive load from the cases and allow participants to concentrate on the simulation scenarios.

The orientation was followed by a 60-minute VR simulation session during which the learner managed 4 cases of tracheostomy emergencies in the virtual setting (see Table 1). Each case was debriefed in-person by a simulation center faculty member with a 1:1 ratio.

**Table 1:** Tracheostomy-related emergency cases.^a^

Tracheostomy emergency cases	
**Case 1**	Chronic tracheostomy, cuff leak
**Case 2**	New tracheostomy, occluded inner cannula
**Case 3**	New laryngectomy, accidental decannulation
**Case 4**	New tracheostomy, false passage

a Details are presented in supplementary e-appendix.

Participants from the small group discussion cohort attended a 60-minute, case-based discussion session facilitated by a different member of our simulation center faculty, with a maximum of 4 learners per session. The cases discussed were identical and are presented in Supplementary e-Appendix 1.

Following each educational intervention, learners from both arms were scheduled for a skills assessment 6-8 weeks after their initial training, which coincided with the start of their subsequent clinical rotation in our training program. Skills were assessed on a novel task trainer to which none of the participants had prior exposure. The assessment sessions were video recorded and graded by 3 independent and blinded graders from unaffiliated institutions. The checklist-based assessment can be found in Supplementary e-Appendix 2, and details regarding design and validation are described below.

Participants in both groups were surveyed before and after their assigned educational intervention regarding attitudes around VR training.

### Survey development

Surveys were developed according to best practices in survey design.^22^ Questions on confidence and competence were adapted from the Perceived Competence Scale (PCS), a previously validated 4-item questionnaire developed through the lens of self-determination theory.^23^^,^^24^ Questions regarding VR experience were adapted from the previously studied Technology Acceptance Model.^25^ Consensus was confirmed with multiple expert faculty from our institution’s Program for Medical Education Innovations and Research. Cognitive interviews were performed, and the surveys were piloted with a validation cohort and iteratively finalized before distribution to participants. All Likert items used a scale from 1-6, where 6 represents the strongest agreement.

### Development of VR modules

Based on literature review of tracheostomy complications, published simulation cases, and tracheostomy emergency guidelines, our group reached expert consensus on 6 cases to include in our tracheostomy emergency curriculum.^6^^,^^10^^,^^26-28^ These scenarios included cuff leak, mucous plugging, accidental decannulation (for new and chronic tracheostomy tubes), malposition in a false passage, and bleeding tracheo-innominate (TI) fistula. We chose a subset of 4 of these cases (Table 1) to develop and pilot in VR simulation based on discussion with the programmers at SimX, a VR medical simulation company. Each of the 4 cases was programmed in iterations based on repeated meetings and piloting feedback from our team. Cases were optimized for implementation with the Oculus Quest 2 head-mounted display. The cases remain available for use within the SimX catalog.

### Development and validation of the checklist assessment tool

Through the lens of the NTSP Tracheostomy Adult Emergency Guidelines, we developed a checklist-based assessment for each of the tracheostomy emergencies via modified Delphi technique, using a panel of experts from pulmonary medicine, critical care, anesthesia, and otolaryngology.^10^^,^^29^ Once stability of the assessment was achieved, we conducted validation testing with a cohort of 3 attending critical care physicians working in a relatively low-volume tracheostomy center with no prior training intervention. The assessments were video recorded and graded by 3 unaffiliated reviewers for calculation of interrater reliability. The results of these assessments were also used to inform sample size calculations and to provide each of the reviewers with approximately 30 minutes of dedicated grader training on the checklists.

### Development of the tracheostomy task trainer

Given the lack of currently available task trainers that could adequately simulate all 6 tracheostomy emergencies in our curriculum, we developed a novel task trainer in consultation with Pulse Medical Demonstration Models. The result was a modular task trainer with 4 anatomical attachments: normal, postlaryngectomy, false passage, and TI fistula. We used this task trainer for final skills assessment given that no study participant had prior experience with this model.

### Statistical analysis

Statistical analysis was performed using Jamovi 2.6.24. To estimate a sample size, we used data from the checklist assessment in our validation cohort. We calculated that 18 fellows would be required to achieve 80% statistical power to show an absolute between-group difference of 4 points on the assessment, assuming a standard deviation of 3. All mean values are reported with standard deviations. For validation data, we used Fleiss’ kappa to determine interrater reliability between 3 independent graders assessing nominal variables. For survey results, we used Wilcoxon signed-rank tests to examine the differences in means for nonparametric Likert data among paired participants. For assessment results, we used independent samples *t*-tests to examine the statistical significance of the differences between means for 2 learner groups.

### Ethics and funding

Data from this study are reported only from trainees who provided informed consent for their educational data to be collected as part of the Research on Medical Education Outcomes database, an institutional review board–approved Resident Medical Education Research Registry.^30^ Data were de-identified prior to analysis. This study was funded via the Association of Pulmonary & Critical Care Program Directors (APCCMPD), American College of Chest Physicians (CHEST), and American Thoracic Society (ATS) Education Research Award. No funding was provided by any medical simulation, technology, or equipment company (including SimX), and the authors have no financial interest in any software produced during this study.

## Results

### Cost and resource comparison

Detailed economic analysis is outside the scope of this study due to rapidly changing costs of associated technology and variable cost of faculty resources. Implementation of the VR simulation program required a compatible headset (approximately $300), licensing fees for the VR software (approximately $3000 annually), and the presence of simulation faculty in a 1:1 ratio with students. In the future, debriefing may become automated via artificial intelligence, but that feature is not yet available. Discussion group programming required the presence of simulation faculty in a 1:4 ratio with learners and a schedule conducive to gathering multiple learners at once.

### Checklist assessment validation

Of 26 possible points on the assessment checklist, the validation cohort scored an average (SD) of 12.33 (1.50). For a more conservative measure of standard deviation likely to reflect a larger population of fellows, we doubled this standard deviation estimate in calculation of our sample size. The validation cohort generated 87 checklist variables, which were either performed or not performed. In review by 3 independent graders, there was 90.8% agreement with a Fleiss’ kappa of 0.875, indicating almost perfect agreement.^31^

### Fellow performance

Of 27 eligible critical care fellows, 19 fellows were recruited and consented to the use of their educational data in this study (Table 2). Fellows participated in a total of 76 simulated tracheostomy emergencies. There were 10 fellows in the VR arm and 9 fellows in the small group arm.

**Table 2:** Demographics of participating fellows.

Fellow characteristics	**VR group (*n*** = **10)**	**Small group (*n***= **9)**
**First-year, No.**	5	1
**Second-year, No.**	4	5
**Third-Year, No.**	1	3
**Male, No.**	5	8
**Female, No.**	5	1
**Age, mean ± SD**	32.44 ± 1.94	33.40 ± 3.55
**Previous VR experience for gaming, No.**	1	1
**Previous VR experience for medical simulation, No.**	1	1
**Affinity for new technology, Likert mean ± SD**	4.00 ± 1.49	4.88 ± 1.36

Abbreviation: VR, virtual reality.

**Figure 1:**
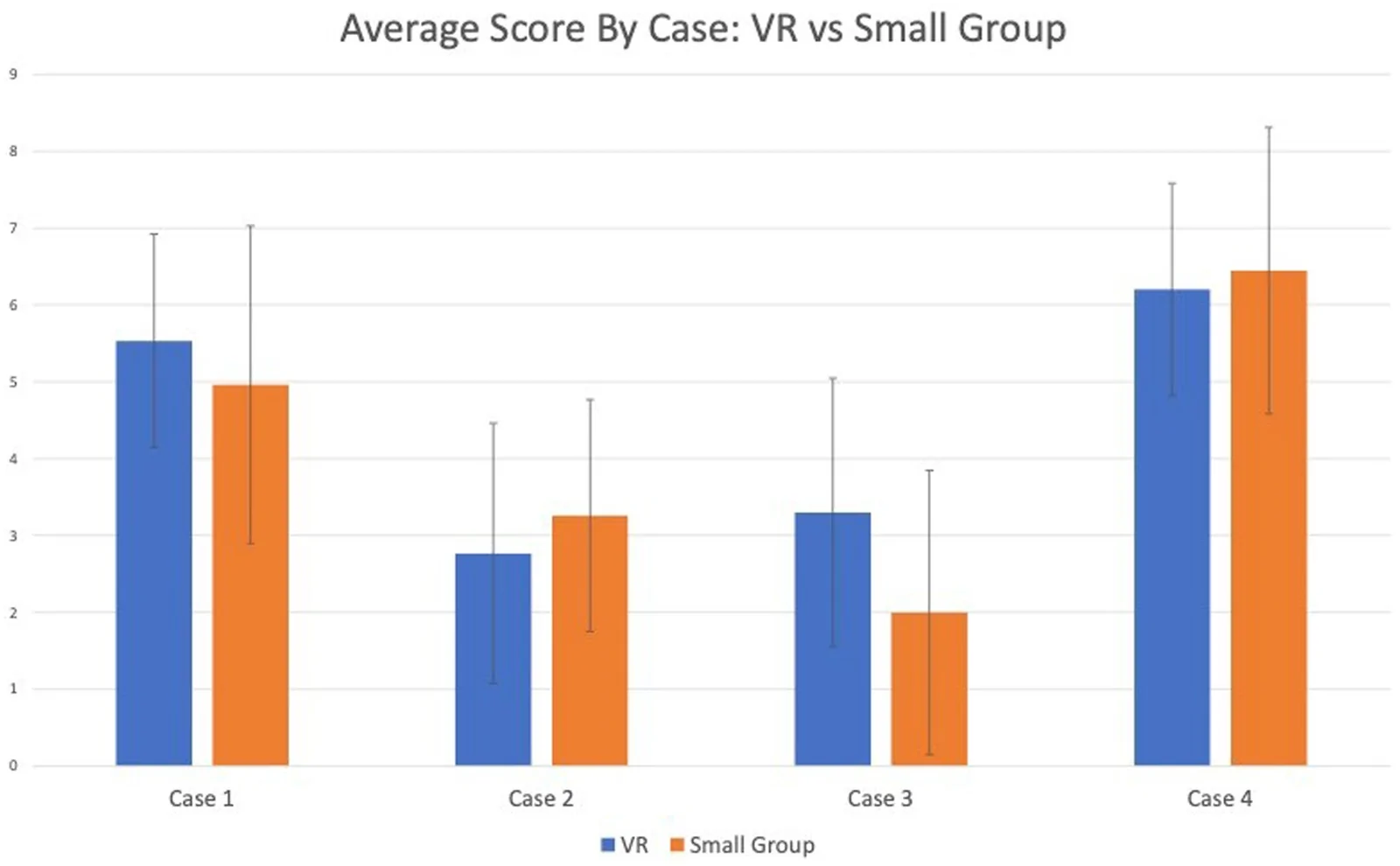
Fellow performance by case. The bar graph shows performance on each case with error bars representing standard deviation. The overlapping error bars show no statistically significant difference between groups for any case. Abbreviation: VR, virtual reality.

Of a total possible 26 points on the checklist assessment, fellows in the VR group scored an average (SD) of 18.03 (3.39) compared to the small group score of 16.96  (4.41) (*P* = .558). Fellows’ performance on the individual cases is presented graphically in Figure 1. No statistically significant difference in performance was detected for any of the 4 piloted cases.

### Survey results

Pre- and postsurvey responses from both study groups representing the PCS are shown (Table 3). Confidence increased significantly for all 4 scale components in the VR cohort and 2 of the 4 scale components in the small group cohort. When asked to rank agreement with the statement, “This curriculum met my needs with respect to the knowledge and skills required to correctly manage tracheostomy emergencies,” the VR group responded with a mean (SD) of 5.0 (1.05) compared to a mean (SD) of 4.63 (1.06) in the control group (*P* = .459).

**Table 3:** Fellow scores on perceived competence scale.

	VR group, mean ± SD			Small group, mean ± SD		
Likert item^a^	Pre-intervention	Postintervention	*P*-value	Pre-intervention	Postintervention	*P*-value
**I feel confident in my ability to recognize and manage tracheostomy emergencies.**	2.9 ± 1.20	4.43 ± 0.95	.008	3.0 ± 1.07	3.88 ± 0.64	.065
**I am capable of recognizing and managing tracheostomy emergencies.**	2.5 ± 0.85	4.42 ± 0.63	.005	2.88 ± 0.83	3.75 ± 0.71	.086
**I am able to correctly manage common tracheostomy emergencies now.**	2.5 ± 0.85	4.1 ± 0.88	.008	2.38 ± 1.06	3.5 ± 0.53	.048
**I feel able to meet the challenge of correctly managing tracheostomy emergencies.**	2.4 ± 0.84	4.1 ± 0.99	.005	2.25 ± 1.16	3.75 ± 0.46	.021

a Likert scale from 1-6, where 6 represents the strongest agreement.

Survey responses based on the Technology Assessment Model are reported for fellows who participated in the VR experience (Table 4). There was an overall high level of acceptance among fellows for the VR curriculum.

**Table 4:** Fellow score on technology assessment model (for VR group).

Likert item^a^	Mean ± SD
**Interactions with the VR environment seemed natural**	3.7 ± 1.16
**I was involved in the virtual environment experience.**	5.0 ± 0.94
**I was able to concentrate on the assigned tasks or required activities rather than on the mechanisms used to perform those tasks or activities.**	4.7 ± 1.42
**I find VR easy to use.**	4.3 ± 0.95
**I find VR useful.**	4.9 ± 0.74
**I intend to use VR in the future.**	4.5 ± 1.08

Abbreviation: VR, virtual reality.

a Likert scale from 1-6, where 6 represents the strongest agreement.

## Discussion

There was no significant difference in fellow performance after VR training vs small group training overall or for any individual simulation case. This supports noninferiority for the VR intervention. Our group has suggested in the past that research around educational technology should not focus solely on the imperative to “prove” that one educational strategy is superior to another but should also investigate how to best leverage the differences between approaches.^19^^,^^32^ If small group discussion and VR simulation yield similar educational outcomes, what are the differences between them in scalability, reproducibility, and resource requirements? For instance, VR simulation can facilitate distance learning, and learners can repeat training exercises as required without the presence of additional faculty. However, small group discussion requires a smaller upfront investment. Traditional mannequin simulation requires its own unique faculty ratios and resource investments. Future research should focus on identifying the best learner populations and educational settings suited to each intervention, allowing a tailored approach aligned with the needs of each individual educational context.

In addition to comparing learning modalities, the results of this study reinforce findings throughout the literature that highlight a competency gap in the management of tracheostomy emergencies. Perceived competence and confidence before training was low among fellows. Performance was lower than expected among the validation cohort of critical care attendings, and even after dedicated training, performance showed significant room for improvement in both study groups. Interestingly, while learners in the VR group experienced significant increases in all PCS items, small group participants only achieved statistically significant increases in 2 of 4 PCS domains. It should be noted that this study was not specifically powered to detect differences in PCS scores, and future studies should compare changes in confidence to changes in performance to better explain why these outcomes may not occur in parallel. Future studies should also further characterize the learning curve for these skills over time as learners achieve proficiency and expertise, along with the retention or deterioration of skills. For instance, future studies could assess for differences in performance after deliberate practice using each learning modality to assess any performance differences that may become apparent after recurrent learning opportunities and continued feedback.^33^

In the use and characterization of our checklist-based assessments, we collected validation evidence that could support the future use of these assessments in similar settings. Considering Messick’s 5-source validity framework, we demonstrated content and response process validity among a group of experts in the field.^34^^,^^35^ We also demonstrated evidence of internal structure via excellent interrater reliability among 3 independent and unaffiliated graders. Future studies should collect evidence regarding relationships with other variables and consequences, as these aspects of validity evidence were outside the scope of this study.

While supporting VR simulation as a feasible approach to tracheostomy-related emergency education, this study does have several limitations. The sample population was relatively small and limited to a single, urban academic medical center. While conclusions can likely be generalized to other large academic centers in the United States, findings may not apply to other training settings. Presumably because of small sample size, random assignment was not perfect, and the small group participants included more experienced fellows than the VR group.

As an additional caveat, we chose to compare educational outcomes from a simulation modality to small group discussion. A more direct comparison may have been to compare VR simulation to mannequin simulation, but in this case, one study group would have had prior exposure and practice with the same task trainer used in the final assessment. We felt this would diminish the validity argument for our checklist assessment and our primary outcome. For the same reason, we avoided a pre-intervention task trainer skills assessment, which may have been helpful to characterize pre-intervention skills but which we felt was not worth the compromise in study validity given the use of a randomized population of learners with similar baseline experience. Finally, we avoided the use of a no-intervention control arm as our team felt providing one group with no education would be unethical.

With data from this pilot study showing feasibility, future studies could include larger and more diverse sample populations, test pre-intervention skills, and measure more robust outcomes, including direct workplace observation and clinical patient outcome data. As a final note, we are still in the development phase with SimX for a case of accidental decannulation of a chronic tracheostomy and just recently completed development for a TI fistula case. These cases are therefore not assessed in the current study but could be assessed in future studies of this technology.

In this randomized educational intervention, fellow performance on a checklist-based simulation assessment showed no significant difference after exposure to immersive VR simulation vs small group discussion. Future research must be done to determine the best learner populations and educational settings suited to each educational strategy.

## Supplementary Material

aapag023_Supplementary_Data

## Data Availability

This article has a data supplement, which is accessible from this article’s home page.
