# Supplementary material for: Virtual reality simulation for tracheostomy emergencies: a randomized educational intervention
Source: ATS Sch. 2026 May 23;7(3):210–6. doi: 10.1093/atsscholar/aapag023 (PMC13519323; doi:10.1093/atsscholar/aapag023)
Supplement: Supplementary file 1 [file aapag023_supplementary_data.zip › ATS VR e-Appendix 1.docx]

**Supplementary e-Appendix 1: Tracheostomy-Related Emergency Cases**

Case 1: Chronic Tracheostomy with Cuff Leak

The learner is called emergently to the medical ICU to evaluate a 70-year-old male with ARDS requiring full mechanical ventilatory support (FIO2 50%, PEEP 10 cm H2O, RR 12, TV 6 ml/kg IBW). The nurse reports that he had been stable until a few minutes ago when his oxygen saturation began to decrease, and the ventilator started alarming for low pressure and low tidal volume. The nurse has taken the patient off the ventilator and is bagging the patient via the tracheostomy with a bag valve mask. You hear a noise over his neck with each delivered breath. The nurse shows you a picture of the ventilator screen taken right before conversion to bagging. The patient is unresponsive on a Propofol infusion.

The learner is provided with pictures of a ventilator showing a difference in delivered and expired tidal volume, and a volume tracing showing a leak pattern. An audio file of a cuff leak is played on the simulator.

Initial State:

The patient is in respiratory distress, using accessory muscles.

RR 36, BP 150/90, P 120, SpO2 80%.

A tracheostomy tube is in place with a torn cuff.

Nurse response to questions:

When: Percutaneous tracheostomy performed 17 days ago.

Why: Prolonged endotracheal intubation. (Had been an easy intubation with hyper-angulated blade)

What: Shiley 8.0 tracheostomy with inner cannula

Expected management:

1) The learner calls for airway assistance if the learner is not an airway expert
2) Initiates supplemental oxygen via face mask or BVM (must deflate cuff first)
3) The learner identifies a cuff leak (clues: leak sound, tidal volume measurement, volume waveform on ventilator)
4) The learner attempts to re-inflate the cuff
5) The learner asks when the tracheostomy was placed
6) The learner replaces the tracheostomy using an obturator, and places a new inner cannula
7) The learner manages the airway without intubating from above

Case 2: New Tracheostomy with Occluded Inner Cannula due to Mucous Plug

The learner is called for an airway emergency in the radiology department. The nurse states that the patient is a 70-year-old male with ARDS requiring full mechanical ventilatory support (FIO2 50%, PEEP 10 cm H2O, RR 12, TV 6 ml/kg IBW) who has been transported to radiology for a chest CT. He has been adequately sedated with a Propofol infusion. The patient had just been transferred to the CT scanner table and reattached to the ventilator. Upon reattachment, the high-pressure alarm activated, and the oxygen saturation dropped to 80%. The nurse states that the patient was then removed from the ventilator and he/she started bagging with 100% oxygen via connection to the tracheostomy tube which appears to be in its usual position. The nurse reports difficulty with ventilation.

Initial State:

The patient is in respiratory distress. Unresponsive to stimuli. Using accessory muscles.

RR 36, BP 150/90, P 120, SpO2 80%.

A tracheostomy tube with an occluded inner cannula is in place.

Nurse response to questions:

When: Percutaneous tracheostomy performed 2 days ago.

Why: Prolonged endotracheal intubation. (Had been an easy intubation with hyper-angulated blade)

What: Shiley 8.0 tracheostomy with inner cannula

Expected management:

1) The learner calls for airway assistance if the learner is not an airway expert
2) Initiates supplemental oxygen via face mask or BVM (must deflate cuff first)
3) The learner attempts to pass a suction catheter through the tracheostomy, and is unable pass through the inner cannula
4) The learner removes the occluded inner cannula
5) The learner replaces the inner cannula and bags the patient via the tracheostomy
6) The learner manages the airway without intubating from above

Case 3: New Laryngectomy, Accidental Decannulation

The learner responds to an airway emergency called to the radiology department. Upon arrival, the learner encounters a 70-year-old male with a completely decannulated tracheostomy tube. A nurse is bagging the patient with 100% oxygen over the mouth and nose. The patient has been well sedated with a Propofol infusion and had been receiving full ventilatory support (FIO2 50%, PEEP 10, RR 12, TV 6 ml/kg IBW). He had been transported to radiology from the surgical ICU for a CT Neck and Chest. During transfer of the patient from the stretcher to the CT table, the tracheostomy tube became dislodged and is completely out of the stoma (now placed on the patient’s neck). He remains adequately sedated with a Propofol infusion.

Initial State:

The patient is in respiratory distress. Unresponsive to stimuli. Using accessory muscles.

RR 36. BP 150/90, P 120, SpO2 80% and rapidly decreasing.

A tracheostomy tube is completely decannulated, now placed on the patient’s neck.

Nurse response to questions:

When: Laryngectomy performed 2 days ago.

Why: Laryngectomy was performed for laryngeal carcinoma, tracheostomy tube was placed because the patient was unable to liberate from the ventilator upon completion of the surgery.

What: Shiley 8.0 tracheostomy with inner cannula

Expected Management:

1) The learner calls for airway assistance if the learner is not an airway expert
2) Initiates supplemental oxygen over the laryngectomy stoma, using a pediatric facemask or bagging with a small laryngeal mask airway
3) The learner asks why the tracheostomy was placed, recognizing that this is a laryngectomy, and the upper airway cannot be used to oxygenate or ventilate
4) The learner replaces the tracheostomy tube using an obturator, and replaces the inner cannula
5) The learner ventilates the patient via the tracheostomy tube
6) The learner should not attempt to ventilate or intubate from above

Case 4: New Tracheostomy in a False Passage

The learner responds to an airway emergency in the Medical ICU for a 65-year-old obese patient in severe respiratory distress. The patient has ARDS, has been well sedated with a Propofol infusion, receiving full ventilatory support (FIO2 50%, PEEP 10, RR 12, TV 6 ml/kg IBW.). The nurse reports that the patient’s distress started just a few minutes ago, after completion of a chest x-ray. The nurse noted that the high-pressure alarm was activated on the ventilator, so she/he took the patient off the ventilator and started bagging via the tracheostomy with 100% oxygen. The tracheostomy does not appear to be obviously decannulated, but the nurse reports difficulty bagging.

Initial State

The patient is in respiratory distress. Unresponsive to stimuli. Using accessory muscles.

RR 36. BP 150/90, P 120, SpO2 80%.

A tracheostomy tube is in place, without apparent mispositioning.

After 30 seconds to allow learner troubleshooting, the neck is inflated on the task trainer with the attached air bulb

Nurse response to questions:

When: Percutaneous tracheostomy performed 2 days ago.

Why: Prolonged endotracheal intubation. (Had been an easy intubation with hyper-angulated blade and video-laryngoscope)

What: Shiley 8.0 tracheostomy with inner cannula

Expected Management:

1) The learner calls for airway assistance if the learner is not an airway expert
2) Initiates supplemental oxygen via face mask or BVM (must deflate cuff first)
3) The learner attempts to pass a suction catheter through the tracheostomy, and is unable to pass farther than about 2 inches
4) The learner removes the inner cannula as a troubleshooting step (in case of occlusion)
5) The learner deflates the cuff as a troubleshooting step (in case it is eccentrically pushed against the tracheal wall)
6) Once neck distention is noted, the learner instructs the nurse to stop ventilating via the tracheostomy, as it is causing subcutaneous crepitus and is apparently in a false passage
7) The learner determines the age of the tracheostomy
8) The learner removes the old tracheostomy tube and covers the stoma
9) The learner performs orotracheal intubation after pre-oxygenation from above
