# Supplementary material for: Virtual reality simulation for tracheostomy emergencies: a randomized educational intervention
Source: ATS Sch. 2026 May 23;7(3):210–6. doi: 10.1093/atsscholar/aapag023 (PMC13519323; doi:10.1093/atsscholar/aapag023)
Supplement: Supplementary file 1 [file aapag023_supplementary_data.zip › ATS VR e-Appendix 2.docx]

**Supplementary e-Appendix 2**

Case 1: Chronic Tracheostomy with Cuff Leak

Learner ID: ____________

Date: __________________

Grader: ________________

Time to case completion: ______ min, _____ sec

| Task | Completed |
| --- | --- |
| Calls for airway assistance (Residents or APPs) |  |
| Places oxygen over the face and/or bags patient via face mask |  |
| Recognizes presence of cuff leak (includes but not limited to auscultation, review of ventilator waveforms/alarms/tidal volumes, and/or verbalizing thought process) |  |
| Attempts to reinflate tracheostomy cuff |  |
| Asks when the tracheostomy was placed (or looks at sign on wall) |  |
| Replaces tracheostomy using obturator/bougie |  |
| Replaces tracheostomy WITHOUT using obturator/bougie |  |
| Removes obturator/bougie |  |
| Manages airway WITHOUT intubating from above |  |

Deviations from expected management:

__________________________________________________________________________________________________________________________________________________________________________________________________________________

Case 2: New tracheostomy with Occluded Inner Cannula

Learner ID: ____________

Date: __________________

Grader: ________________

Time to case completion: ______ min, _____ sec

| Task | Completed |
| --- | --- |
| Calls for airway assistance (Residents or APPs) |  |
| Places oxygen over the face and/or bags patient via face mask (must deflate trach cuff first) |  |
| Removes inner cannula |  |
| Attempts to pass soft suction catheter through OLD tracheostomy tube, or confirms RN has already attempted |  |
| Bags patient via tracheostomy (must remove old inner cannula, can bag with or without new inner cannula) |  |
| Manages airway WITHOUT intubating from above |  |

Deviations from expected management:

__________________________________________________________________________________________________________________________________________________________________________________________________________________

Case: New Laryngectomy with Accidental Decannulation

Learner ID: ____________

Date: __________________

Grader: ________________

Time to case completion: ______ min, _____ sec

| Task | Completed |
| --- | --- |
| Calls for airway assistance (Residents or APPs) |  |
| Places oxygen over the laryngectomy stoma |  |
| Asks why the tracheostomy was placed (e.g. indication for placement, or specific surgical procedure performed) |  |
| Replaces tracheostomy tube using obturator/bougie |  |
| Replaces tracheostomy tube WITHOUT using obturator/bougie |  |
| Replaces tracheostomy tube with an endotracheal tube (i.e. inserts ETT through stoma) |  |
| Removes obturator/bougie |  |
| Performs bag ventilation or connects to ventilator via tracheostomy |  |

Deviations from expected management:

___________________________________________________

___________________________________________________

___________________________________________________

Case: New Tracheostomy with Displacement into False Passage

Learner ID: ____________

Date: __________________

Grader: ________________

Time to case completion: ______ min, _____ sec

| Task | Completed |
| --- | --- |
| Calls for airway assistance (Residents or APPs) |  |
| Places oxygen over the face and/or bags patient via face mask (must deflate tracheostomy cuff first) |  |
| Instructs RN to stop ventilating via tracheostomy |  |
| Removes inner cannula |  |
| Attempts to pass suction catheter through OLD tracheostomy tube, or confirms RN has already attempted |  |
| Deflates tracheostomy cuff as a troubleshooting step (not only for removal of tracheostomy tube) |  |
| Asks when the tracheostomy was placed |  |
| Removes tracheostomy tube |  |
| Asks for or performs bag valve mask ventilation from above with occlusion of the stoma prior to reintubation |  |
| Performs orotracheal intubation or requests orotracheal intubation |  |

Deviations from expected management:

__________________________________________________________________________________________________________________________________________________________________________________________________________________
